# Synergy between Multiple Microtubule-Generating Pathways Confers Robustness to Centrosome-Driven Mitotic Spindle Formation

**DOI:** 10.1016/j.devcel.2013.12.001

**Published:** 2014-01-13

**Authors:** Daniel Hayward, Jeremy Metz, Claudia Pellacani, James G. Wakefield

**Affiliations:** 1 Biosciences, College of Life and Environmental Sciences, University of Exeter, Stocker Road, Exeter EX4 4QD, UK; 2 Istituto Pasteur-Fondazione Cenci Bolognetti, “La Sapienza” Università di Roma, P.le A. Moro 5, 00185 Roma, Italy

## Abstract

The mitotic spindle is defined by its organized, bipolar mass of microtubules, which drive chromosome alignment and segregation. Although different cells have been shown to use different molecular pathways to generate the microtubules required for spindle formation, how these pathways are coordinated within a single cell is poorly understood. We have tested the limits within which the *Drosophila* embryonic spindle forms, disrupting the inherent temporal control that overlays mitotic microtubule generation, interfering with the molecular mechanism that generates new microtubules from preexisting ones, and disrupting the spatial relationship between microtubule nucleation and the usually dominant centrosome. Our work uncovers the possible routes to spindle formation in embryos and establishes the central role of Augmin in all microtubule-generating pathways. It also demonstrates that the contributions of each pathway to spindle formation are integrated, highlighting the remarkable flexibility with which cells can respond to perturbations that limit their capacity to generate microtubules.

## Highlights

• Chromosome-driven MT generation exists in embryos and is dependent on D-HURP
• Centrosome disruption results in cytoplasmic aMTOCs, driving spindle formation
• Augmin generates MTs from centrosomal, chromatin, and aMTOC MTs indiscriminately
• Reducing one pathway synergistically increases MT growth of the remaining pathways

## Graphical Abstract

**Figure:**
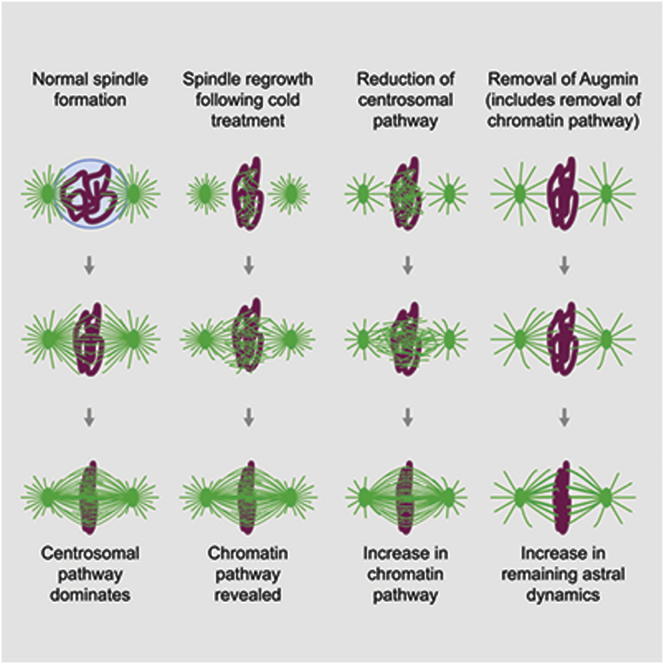

## Teaser

Hayward et al. explore the limits within which *Drosophila* embryonic mitotic spindles can form. By disrupting temporal control of microtubule generation and interfering with microtubule generation pathways, they uncover complementary routes to spindle formation, demonstrating that removal of one microtubule-generating pathway results in a compensatory increase from the others.

## Introduction

During mitosis, microtubules (MTs), dynamic polymers of α and β tubulin, are nucleated in sufficient number so that they form a bipolar spindle apparatus, generating the force required for accurate alignment and segregation of duplicated chromosomes. Work in different model organisms has shown that the route to spindle formation can vary; for example, the MTs that constitute spindles in *Xenopus* egg extracts are initially nucleated by condensed chromatin (Heald et al., 1996), the assembly of the *Drosophila* early embryonic spindle is regarded as centrosome directed (Brust-Mascher et al., 2009), while mammalian oocytes generate MTs in the cytoplasm that gradually coalesce to bipolarity (Brunet et al., 1998, Schuh and Ellenberg, 2007). In addition, MT-dependent MT generation, catalyzed by the Augmin complex, provides an additional pathway that contributes to overall spindle MT density (Goshima et al., 2007, Goshima et al., 2008, Uehara et al., 2009, Wainman et al., 2009). Given that most animal mitotic cells possess centrosomes and chromatin within a substantial cytoplasm and that Augmin is functionally conserved, one important question is whether all individual MT-generating pathways coexist in a single cell. If they do, and if their functions are integrated, it could explain why mature spindles are so robust when challenged with physical, genetic, and chemical perturbations.

Although previous research has addressed the relationship between centrosomal and chromatin-generated MTs (Heald et al., 1997, Khodjakov et al., 2000, Mahoney et al., 2006, Maiato et al., 2004) these studies were undertaken prior to discovery of Augmin (Goshima et al., 2007), and in tissue culture cells. The relationship between all the major defined MT generating pathways and their significance within a developmental context therefore remains unclear. Here, we use the *Drosophila* syncytial blastoderm embryo in order to comprehensively address how a mitotic spindle forms. This tissue, in which many hundreds of mitotic spindles form simultaneously in a common cytoplasm, allows manipulation of MT-generating pathways not only through genetics but also through immediate inactivation of proteins facilitated by interfering antibody injections (see, for example, Brust-Mascher et al., 2009, Conduit et al., 2010). We have combined these advantages with a live cold-treatment assay that allows mature embryonic mitotic spindles to be deconstructed and rebuilt and with the development and implementation of image analysis software that allows quantitative data to be extracted simultaneously from multiple spindles. Our results demonstrate that MTs can be generated in this system by mitotic chromatin, in addition to centrosomes, using a molecular pathway dependent on the *Drosophila* homolog of the spindle assembly factor, HURP. By disrupting the accumulation of two pericentriolar material (PCM) proteins, DSpd-2 and Centrosomin (Cnn), to the centrosome, we also find that *Drosophila* embryos can form bipolar spindles from multiple cytosolic acentrosomal MT organizing centers (aMTOCs). We show that all these routes to spindle formation are supplemented by Augmin-generated MTs; inactivation of Augmin abrogates chromatin-generated and aMTOC-dependent MTs and substantially delays and reduces astral MT input. We also demonstrate that integration does, indeed, exist between pathways. A reduction in centrosome-generated MTs leads to an increased rate of MT nucleation around chromatin, while a loss of chromatin- or Augmin-dependent MT nucleation increases the growth rate of remaining astral MTs. We also show that this effect is synergistic. Thus, mitotic MT generation in a cell within a developing organism comprises coordinated inputs from multiple MT-nucleating pathways, providing inherent robustness and flexibility to the mature mitotic spindle.

## Results

### MT Nucleation Occurs from Chromosomes during *Drosophila* Embryonic Mitosis

**Figure 1:**
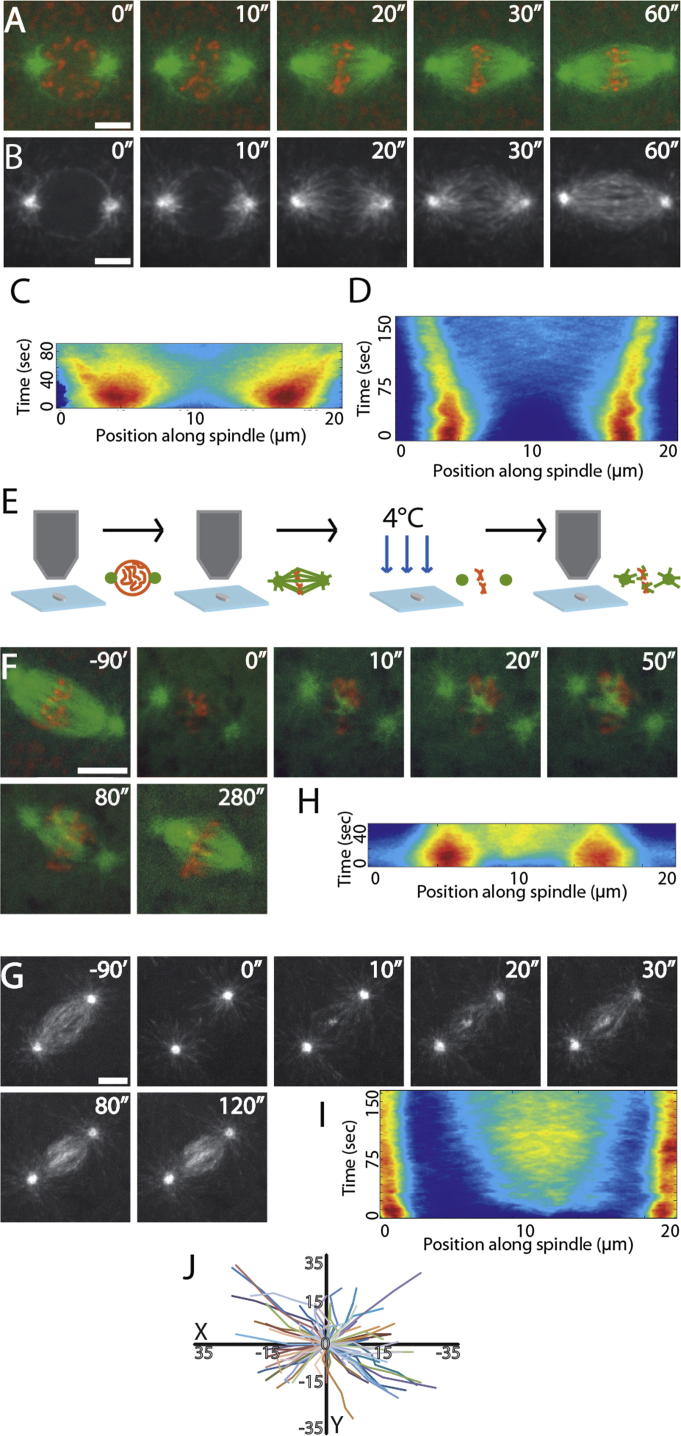
Cold Treatment of *Drosophila* Embryos Reveals MT Nucleation from Chromatin during Mitosis (A and B) Stills from movies of spindle formation in embryos expressing α-Tubulin-GFP (green) and Histone-RFP (red) to visualize MTs and chromatin, respectively (A), or the MT growing plus-end marker EB1-GFP (B). Mitotic spindles unambiguously form in an “outwards-in” manner. (C and D) Composite kymographs of Tubulin-GFP (C) and EB1-GFP (D) with heat-map representations of MT intensity. (E) Schematic diagram of cold-treatment protocol (see Results). (F and G) Stills from movies of spindle reformation in syncytial embryos expressing Tubulin-GFP; Histone-RFP (F) or EB1-GFP (G) following cold treatment. MT regrowth is apparent both at centrosomes and around chromatin. (H and I) Composite kymographs of Tubulin-GFP (H) and EB1-GFP (I) in embryos following cold-treatment recovery, with heat-map representations of MT intensity. (J) Graph showing the initial random directionality of EB1 comets emanating from chromatin on cold-treatment recovery. Scale bars, 5 μm. See also Movies S1 and S2.

We began by exploring whether chromatin-mediated MT nucleation could be visualized during mitotic spindle formation in *Drosophila* syncytial embryos. We subjected embryos expressing either Tubulin-green fluorescent protein (GFP) and Histone-red fluorescent protein (RFP) (to monitor MTs and DNA, respectively) or the MT plus-tip protein EB1-GFP (as a marker of MT plus end growth) to high spatial and temporal resolution imaging, using spinning disc confocal microscopy (Figures 1A and 1B; Movie S1 available online). Within 30 s following nuclear envelope breakdown (NEB) and the influx of Tubulin into the nuclear space, the mitotic spindle had formed, appearing exclusively to do so using an “outwards-in” mechanism directed by the generation and growth of MTs from the centrosomes. To quantify the MT growth, we developed an automated tracking algorithm to extract data regarding Tubulin-GFP and EB1-GFP intensity over time, from multiple spindles within individual embryos (see Experimental Procedures). This confirmed the almost exclusive movement of Tubulin-GFP and EB1-GFP comets from pole to center, a phenomenon that was easily visualized by compressing the data sets into single composite kymographs (Figures 1C and 1D; Figure S1).

We wondered whether the apparent exclusivity of centrosomes as the source of spindle MTs was due to spatiotemporal restriction of pathway activation (i.e., that, during these very fast mitoses, there is insufficient time to activate the pathway required for chromatin-mediated MT nucleation). To explore the potential of embryonic mitotic chromatin to generate MTs, we therefore sought to decouple astral MT nucleation from NEB and chromosome condensation. As a noninvasive approach, we made use of the temperature-sensitive property of MTs. Classic experiments have demonstrated that mitotic spindles are depolymerized by incubation of tissue at 4°C and that their repolymerization can be initiated by rewarming, with no negative effects on chromosome segregation (Inoue, 1952, Inoue, 1964). We therefore adhered individual fluorescent embryos to coverslips and monitored their progression through the cell cycle until they reached metaphase (Figure 1E). They were then immediately placed on ice, left for 90 min, placed back under the microscope objective, and imaged to assay spindle regrowth (Figure 1E). Individual movies in embryos expressing either Tubulin-GFP; Histone-RFP (Figure 1F; Movie S2) or EB1-GFP (Figure 1G; Movie S2) together with composite kymographs (Figures 1H and 1I) strongly suggested that, instead of spindle formation occurring “outwards-in,” MTs were organized into bipolar spindles “inwards-out.” Manual tracking of EB1-GFP comets in this region, immediately following cold treatment, demonstrated random directionality, consistent with nonbiased MT growth from the chromatin (Figure 1J). This chromatin-dependent MT nucleation was not merely a consequence of centrosomes losing MT-nucleating capacity during cold treatment, as the intensity of γ-Tubulin-GFP at the centrosomes was similar between cycling and cold-treated embryos (see Figure S1). We therefore conclude that the molecular pathway responsible for chromatin-driven MT generation is present in *Drosophila* embryos but that the dynamics of its activation in relation to centrosomal nucleation normally precludes or masks its involvement in initial spindle formation.

### The *Drosophila* SAF D-HURP Is Essential for Generating Chromatin-Derived MTs

**Figure 2:**
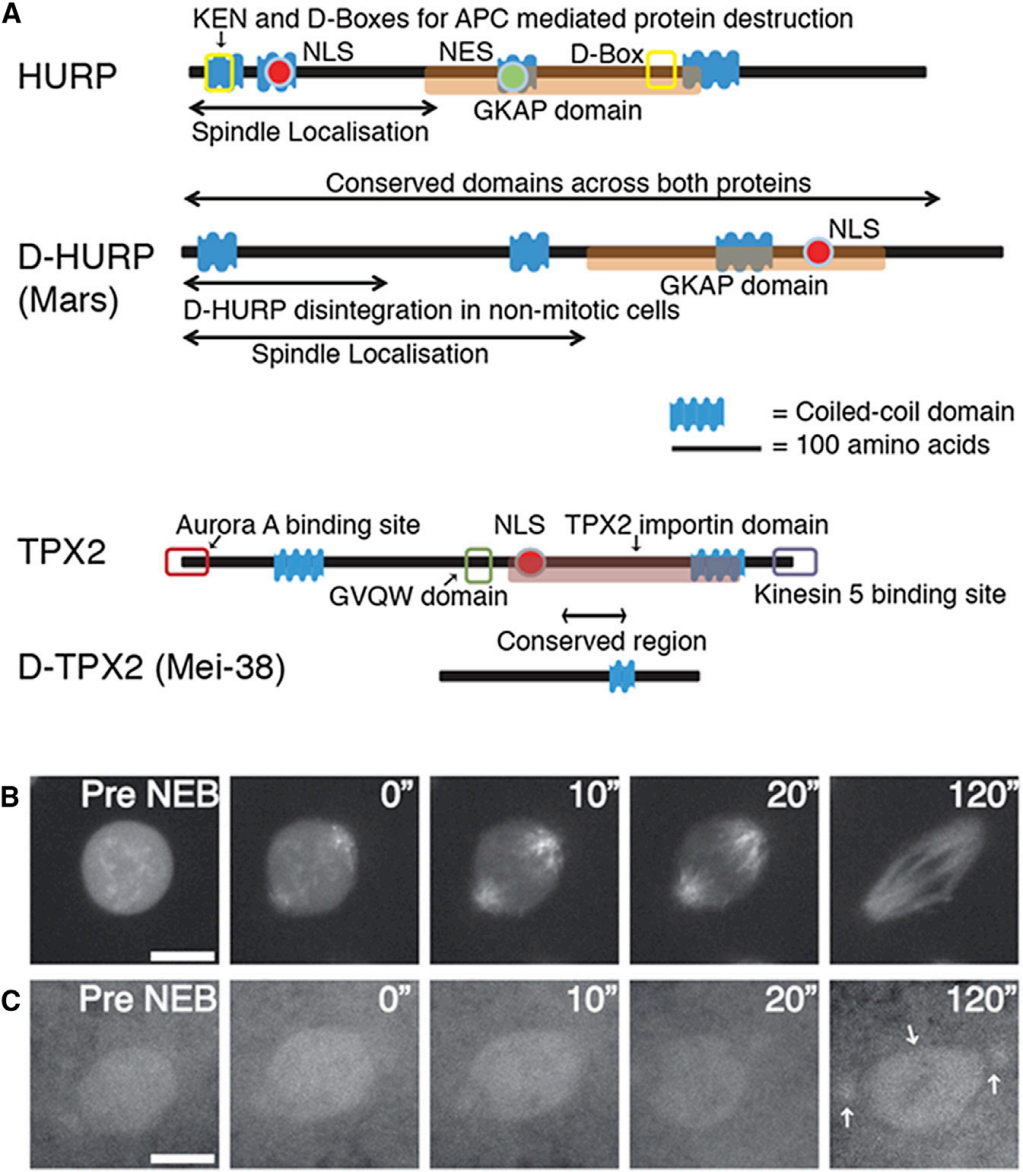
D-HURP and D-TPX2 Are Mitotic MAPs that Dynamically Associate with MTs during *Drosophila* Syncytial Mitoses (A) Schematic representation comparing D-TPX2/Mei-38 and D-HURP/Mars to human TPX2 and HURP. GKAP, guanylate kinase-associated protein; NLS, nuclear localization signal; NES, nuclear export signal. (B and C) Stills from movies of GFP-D-HURP (B) and GFP-TPX2 (C) during embryonic divisions. GFP-D-HURP is nuclear in interphase before accumulating on specifically on spindle, but not astral, MTs following NEB; GFP-TPX2 is nuclear in interphase and localizes weakly to the area of the spindle and to the centrosomes (arrow) during mitosis. Scale bars, 5 μm. See also Movie S3.

To define the molecular basis of chromatin-dependent MT generation in the early embryo, we focused on the *Drosophila* homologs of two chromatin-associated spindle assembly factors (SAFs), HURP (D-HURP/Mars) and TPX2 (D-TPX2/Mei-38/Ssp1) (Figure 2A) (Tan et al., 2008, Wu et al., 2008, Goshima, 2011). As previously reported, GFP-D-HURP was nuclear during the early embryonic interphase (Figure 2B; Movie S3) (Zhang et al., 2009). Upon NEB, it accumulated on spindle MTs as they were generated from centrosomes but not to centrosomes themselves or to astral MTs. Following chromosome segregation, D-HURP relocalized to decondensing chromosomes (Movie S3). GFP-D-TPX2 was also nuclear in interphase but showed only weak localization to the area of the mitotic spindle and centrosomes during mitosis (Figure 2C, arrows).

**Figure 3:**
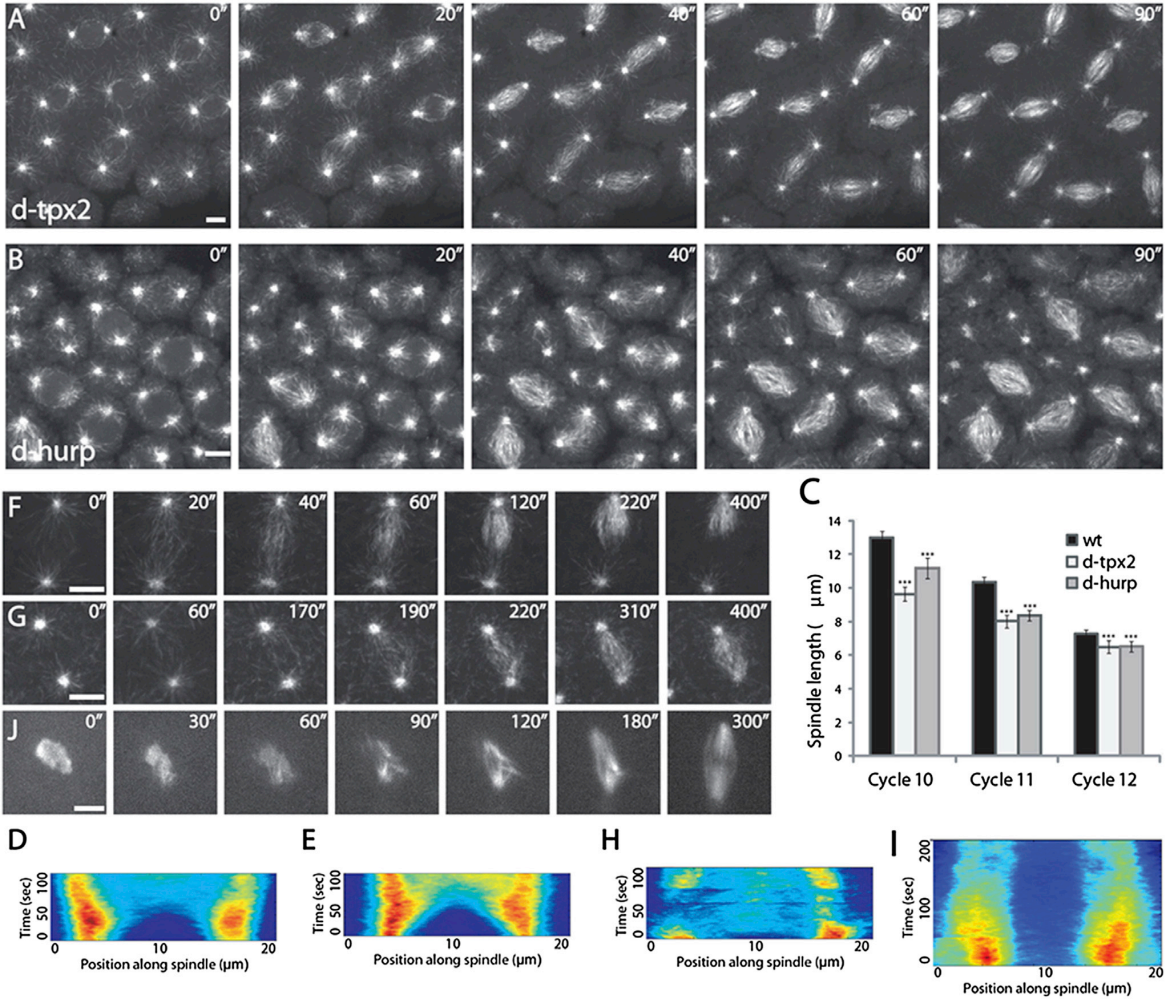
MT Nucleation from Chromatin Is Mediated by D-HURP (A and B) Stills from movies of spindle formation in *d-tpx2 (mei-38*^*1*^*)* (A) and *d-hurp (mars*^*p*^*)* (B) embryos expressing EB1-GFP. Note the presence of abnormal spindles. (C) Bar chart of control (WT), *d-tpx2*, and *d-hurp* spindle length across mitotic cycles 10, 11, and 12. Error bars indicate SEM. (D and E) Composite kymographs of MT nucleation (EB1-GFP) during mitosis in *d-tpx2* (D) and *d-hurp* (E) cycling embryos. (F and G) Stills from movies of spindle reformation in syncytial *d-tpx2* (F) and *d-hurp* (G) mutant embryos expressing EB1-GFP. (H and I) Composite kymographs of MT nucleation (EB1-GFP) during mitosis in *d-tpx2* (H) and *d-hurp* (I) embryos following cold treatment, demonstrating the requirement of D-HURP in chromatin-mediated MT generation. (J) Stills from movies of GFP-D-HURP localization following cold treatment; the protein is now initially present on mitotic chromatin, gradually relocalizing to spindle MTs as they form. Scale bars, 5 μm. See also Movies S4 and S5.

To assess the contributions of D-HURP and D-TPX2 to mitotic spindle formation, we analyzed EB1-GFP dynamics in embryos carrying mutations in the genes encoding these proteins. Flies carrying a null mutation in D-TPX2 (*mei38*^*1*^ flies) are viable and fertile, although they show slightly elevated levels of chromosome missegregation in a variety of tissues (Wu et al., 2008). Similar effects have been reported for the hypomorphic allele of D-HURP, *mars*^*P*^ (Tan et al., 2008). We found that, although the majority of embryonic spindles formed similarly to controls in both these mutants, defective spindle formation was apparent (Figures 3A and 3B; Movie S4). In addition, mature bipolar spindles were consistently significantly shorter than their wild-type counterparts (Figure 3C). Together, these results confirm that both D-HURP and D-TPX2 have roles in embryonic spindle formation.

Next, we analyzed the dynamics of EB1-GFP comets during spindle regrowth in mutant embryos that had undergone cold treatment. In contrast to control embryos, *mei38*^*1*^ mutants failed to form stable bipolar spindles. Although MT regrowth from chromosomes was apparent, these MTs often interacted with the nearest centrosome, without substantial contact to a second, resulting in highly variable composite kymographs (Figures 3F and 3H; Movie S5). Therefore, although D-TPX2 has a clear role in spindle stability following regrowth, it is not required for chromatin-dependent MT generation in the embryo. In contrast, in *mars*^*p*^ mutants following cold treatment, chromatin-dependent MT generation was absent (Figure 3G; Movie S5); composite EB1-GFP kymographs showed no regrowth in the area between the poles (compare Figure 1I and Figure 3I). However, EB1-GFP fluorescence emanating from centrosomes was unaffected (Figure S2). Together, these results clearly demonstrate that, while removal of D-HURP has no effect on centrosomal MT nucleation, D-HURP is essential for chromatin-dependent MT generation.

To further clarify the way in which D-HURP directs MT generation from chromosomes, we assessed whether its subcellular localization differs depending on the dominant MT nucleating pathway used by the embryo. As described earlier, in cycling embryos, D-HURP is nuclear in interphase and associates with MTs as they form from centrosomes early in mitosis (Figure 2B). However, in embryos immediately following cold treatment (i.e., in which chromatin-directed MT generation dominates), D-HURP was exclusively found on mitotic DNA (Figure 3J). Over time, as MTs were generated, it relocalized from chromatin to the spindle MTs. This effect was specific to D-HURP; neither D-TPX2 nor γ-Tubulin, nor the MT-generating complex Augmin was found on mitotic DNA following cold treatment (data not shown). We conclude that D-HURP is an essential effector of chromatin-mediated MT generation in *Drosophila* embryos.

### Augmin Is Essential for Chromosome-Generated MTs and Supplements Astral MT Generation

**Figure 4:**
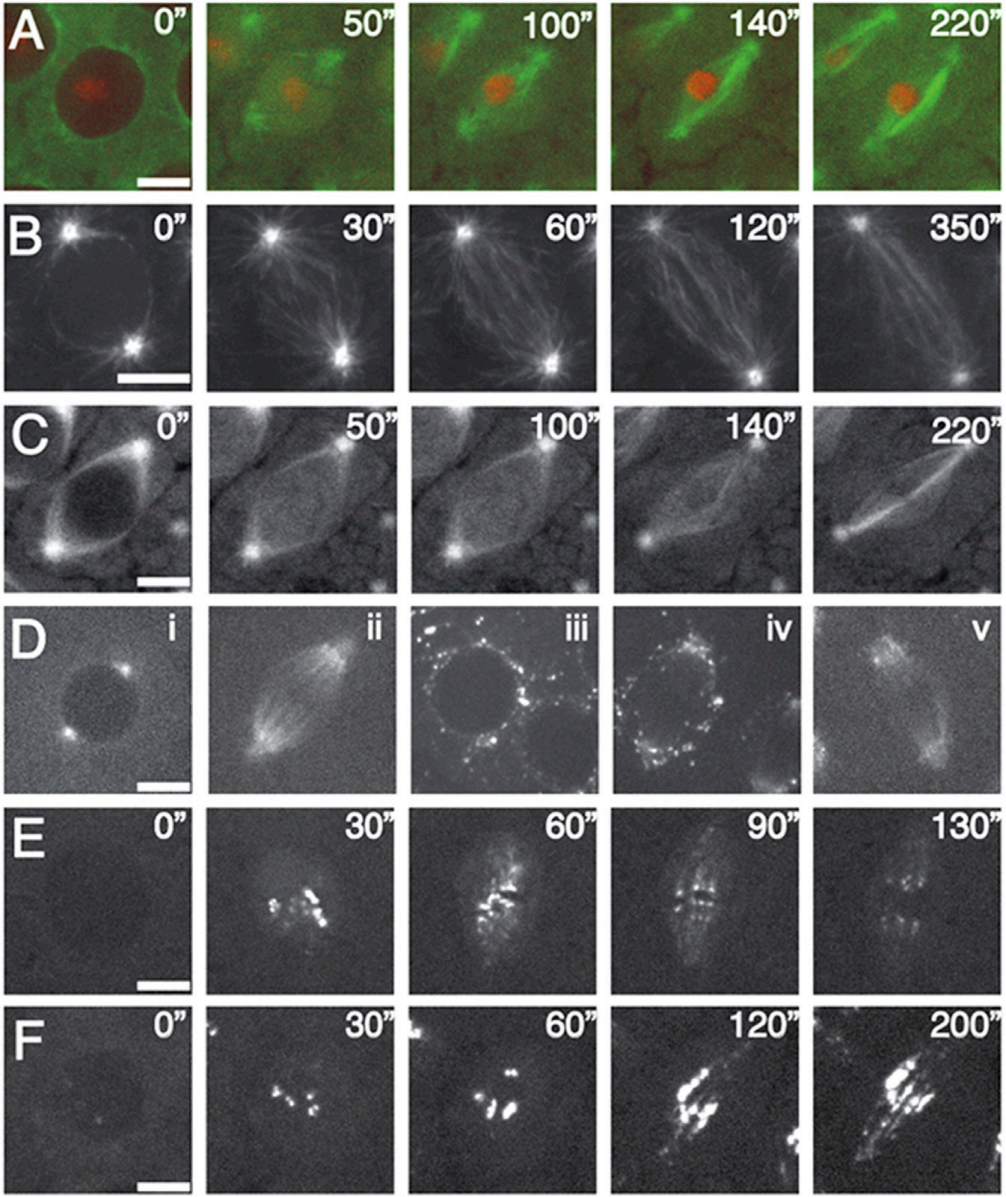
Augmin Is Required for Maintenance of Mitotic Spindle Integrity (A and B) Stills from movies of spindle formation in embryos expressing Tubulin-GFP; Histone-RFP (A) or EB1-GFP (B) injected with interfering antibodies generated against the Augmin subunit, Dgt6. Astral MT and spindle formation are delayed, progressing to weak elongated spindles which arrest. (C) Stills from movies of spindle formation in *msd1*^*ex51*^ embryos expressing Tubulin-GFP. (D) Stills from movies of Msd1-GFP-expressing embryos. Msd1-GFP localizes to MTs in interphase (i) and metaphase (ii). Following injection of anti-Dgt6 antibodies, Msd1-GFP dissipates from all MTs close to the site of injection during interphase (iii) and mitosis (iv). Weak MT localization remains in areas distant (∼50 μm) from the site of injection (v). (E and F) Stills from movies of spindle formation in embryos expressing Rod-GFP (E). Rod localizes to assembling kinetochores in prophase and streams poleward on MT-kinetochore attachment, gradually decreasing with time (30”–120”). In (F), upon injection of anti-Dgt6 antibodies, Rod-GFP streaming is delayed but occurs, signifying the presence of K-fibers. Localization of Rod-GFP to kinetochores and kMTs persists throughout the observation period. Scale bars, 5 μm. See also Movies S6 and S7.

Recent work has shown that MTs are also nucleated from preexisting MTs within the growing spindle—a process that requires both γ-Tubulin and the hetero-octomeric MT-associated protein (MAP) complex, Augmin (Goshima et al., 2007, Goshima et al., 2008, Uehara et al., 2009, Wainman et al., 2009). To define the contribution of such MT-dependent MT nucleation during spindle formation in the early embryo, we removed Augmin function by injecting interfering antibodies generated against the Dgt6 subunit (Bucciarelli et al., 2009). Injection of this antibody into embryos expressing Tubulin-GFP; Histone-RFP or EB1-GFP phenocopied those possessing a mutation in the Msd1 subunit of Augmin (Wainman et al., 2009) and disrupted the association of Augmin with MTs, demonstrating the specificity and effectiveness of this approach (Figures 4A–4D; Movie S6). In cycling embryos injected with anti-Dgt6, spindle formation was delayed with respect to controls. Whereas bipolarity was achieved within ∼30 s in control embryos, spindles in *msd1* mutant embryos, or those injected with anti-Dgt6 embryos, took substantially longer (100–200 s) (Figures 4A–4C; Movie S6). Quantification of EB1-GFP fluorescence around centrosomes following NEB in anti-Dgt6-injected embryos confirmed that MT generation from centrosomes was significantly reduced (Figure S2). Thus, Augmin contributes to astral MT generation during mitosis.

The bipolar spindles that eventually formed upon Augmin inactivation increased in length in comparison to controls and remained arrested at metaphase with fewer MTs, predominantly composed of thick MT bundles (Figures 4A–4C; Movie S6). To assess whether these were kinetochore MTs (kMTs) (i.e., MTs with stable attachments to kinetochores), we injected anti-Dgt6 antibodies into embryos expressing a GFP fusion to the checkpoint protein Rod, comparing its localization to control embryos. Rod is a component of the RZZ complex, which associates with kinetochores early in mitosis and streams poleward along kMTs upon kMT-kinetochore attachments (Basto et al., 2004). Loss of kinetochore-associated Rod facilitates satisfaction of the checkpoint in embryos, therefore allowing progression into anaphase (Basto et al., 2004). In control embryos, Rod-GFP poleward streaming occurred within 30 s following NEB (Figure 4E; Movie S7). In GFP-Rod embryos injected with anti-Dgt6 antibodies, however, although Rod accumulated on kinetochores and streamed along MTs toward the poles, streaming took >60 s to initiate and was abnormal (Figure 4F; Movie S7). Movement of Rod-GFP was stilted and occurred both toward and away from the poles. Consistent with the long spindles and mitotic arrest observed in embryos in which Augmin function is lost, Rod continued to accrue on both kinetochores and MT bundles throughout the period of observation, as opposed to being gradually lost as in control cells. These results demonstrate that Augmin-generated MTs aid, but are not required for, astral MT search and capture of chromosomes, as well as kMT formation. They also show that the MTs generated by Augmin are essential for checkpoint satisfaction and chromosome segregation.

**Figure 5:**
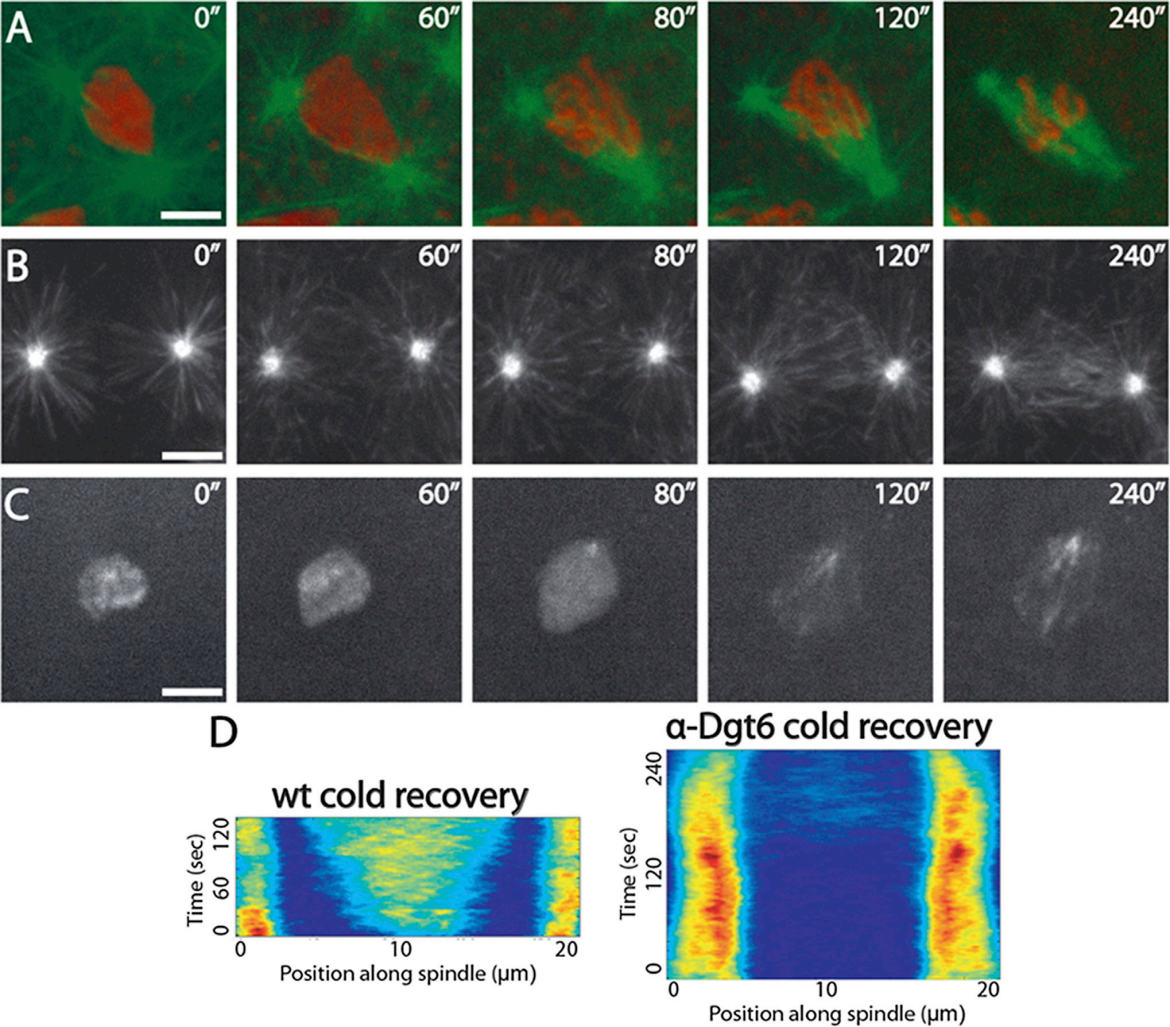
Augmin Is Essential for MT Generation around Chromatin (A and B) Stills from movies of spindle reformation in embryos expressing Tubulin-GFP Histone-RFP (A) or EB1-GFP (B) injected with anti-Dgt6 antibodies. (C) Stills from movies of D-HURP-GFP localization following cold treatment in an anti-Dgt6-injected embryo; localization of D-HURP-GFP is not dependent on Augmin. (D) Composite kymographs of MT nucleation (EB1-GFP) during mitosis in control (WT) and anti-Dgt6-injected embryos following cold treatment; chromatin-dependent MT generation is completely absent upon Augmin disruption. See also Movie S8. Scale bars, 5 μm.

To more precisely define the relationship between Augmin-, centrosomal-, and chromosome-dependent MT nucleation, we injected cold-treated Tubulin-GFP; Histone-RFP- or EB1-GFP-expressing embryos with anti-Dgt6 antibodies (Figures 5A and 5B; Movie S8). We found a complete absence of MT generation in the vicinity of chromosomes, as visualized by composite kymographs (Figure 5D), showing that Augmin, like D-HURP, has an essential role in this process. To investigate the relationship between D-HURP and Augmin in the generation of chromatin-dependent MTs, we sought to determine whether disruption of Augmin affected the dynamic accumulation of D-HURP on mitotic DNA or its relocalization to spindle MTs (Figure 5C). We found no difference in comparison to control cold-treated embryos, demonstrating that D-HURP does not require Augmin-generated MTs to move from chromatin to spindle MTs.

### Centrosome/PCM Disruption Results in aMTOC-Driven Spindle Formation

**Figure 6:**
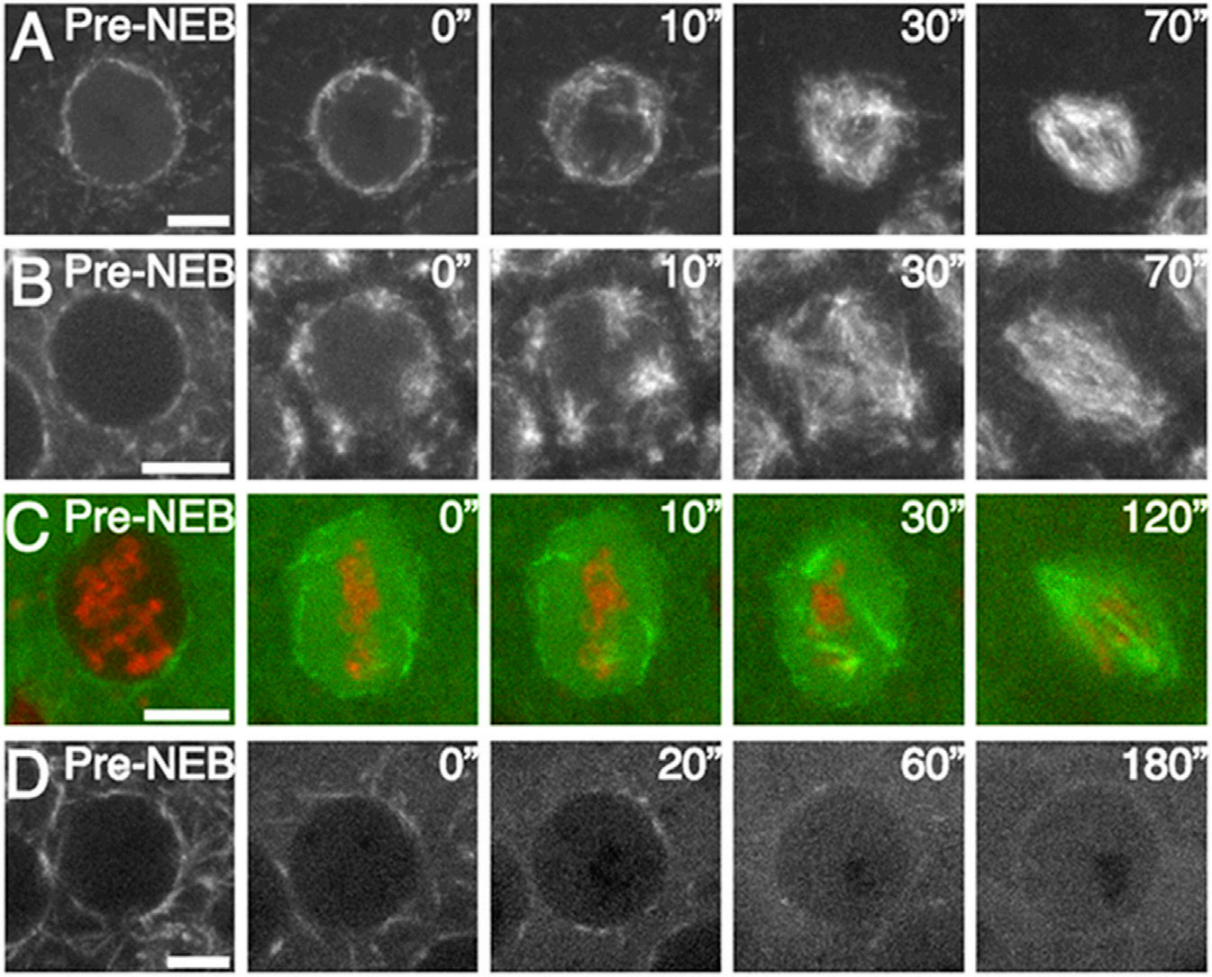
Centrosome/PCM Disruption Leads to Spindle Formation via Augmin-Dependent aMTOCs (A) Stills from movies of spindle formation in an EB1-GFP-expressing *cnn* mutant embryo. (B and C) Stills from movies of spindle formation in EB1-GFP expressing (B) and Tubulin-GFP; Histone-RFP embryos (C) injected with a high concentration of anti-DSpd-antibody. In all cases, spindles form predominantly from cytoplasmic, acentriolar MTOCs. (D) Stills from movies of spindle formation in an EB1-GFP-expressing *cnn* embryo injected with anti-Dgt6 antibodies. MTs are present in interphase, but mitotic aMTOCs do not form. Scale bars, 5 μm. See also Movie S9.

To determine whether chromatin-mediated MT generation is sufficient for spindle formation in the *Drosophila* embryo, we attempted to fully inactivate centrosome-driven MT nucleation. γ-Tubulin is recruited to embryonic centrosomes primarily through the incorporation of Cnn into the PCM, a process that depends cooperatively on two additional proteins, Asterless and DSpd-2 (Conduit et al., 2010). We took two approaches: first, we followed mitotic spindle formation in EB1-GFP-expressing embryos carrying a mutation in the *cnn* gene. Cnn is required for the proper connection between the centriole and the PCM, and although previous fixed analyses of mitotic spindles in this mutant have shown that acentriolar bipolar spindles are capable of forming, they did not clearly define the route to spindle formation (Lucas and Raff, 2007, Megraw et al., 2001). Second, in both EB1-GFP- and GFP-Tubulin; RFP-Histone-expressing embryos, we removed centrosomally nucleated MTs through injection of an interfering antibody raised against DSpd-2, which has previously been shown to displace DSpd-2 and PCM components from the centrosome (Conduit et al., 2010). In all cases, we found that, rather than MTs emanating from centrosomes or chromatin, multiple discrete asters formed in the cytosol upon entry into mitosis (Figures 6A–6C; Movie S9). These ectopic, cytosolic asters continued to expand and cluster and ultimately organized into acentrosomal barrel-shaped spindles. To determine whether the MTs generated by these aMTOCs were dependent on Augmin, we injected anti-Dgt6 antibodies into *cnn* mutant embryos expressing EB1-GFP. We found that inhibition of Augmin completely abrogated aMTOC-driven MT nucleation; by 60 s following NEB, no EB1-GFP comets were visible (Figure 6C; Movie S9). We therefore conclude that, upon disruption of centrosomes, an Augmin-dependent, noncentrosomal pathway can drive MT generation in the early embryo.

### Reducing the Contribution of One MT-Generating Pathway Results in an Increased Contribution from Others

**Figure 7:**
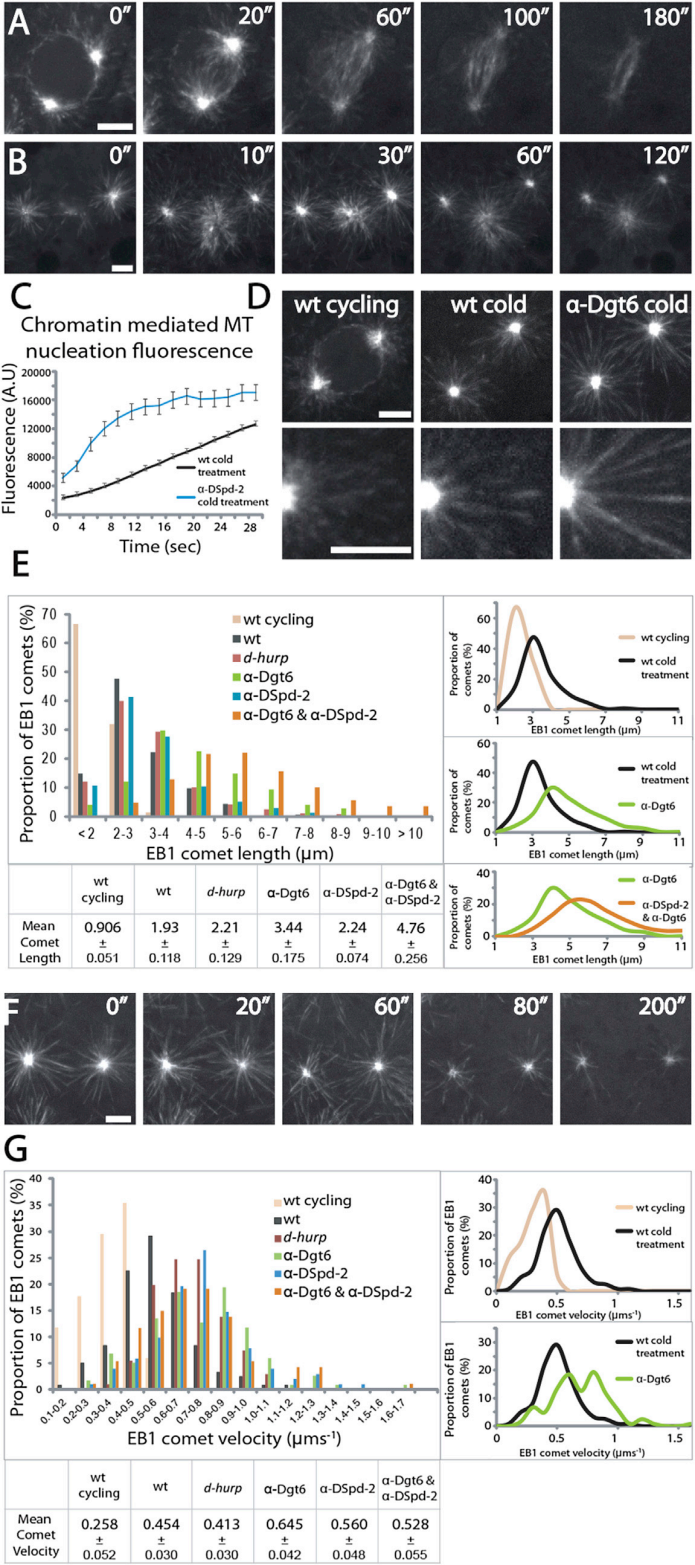
MT-Generating Pathways Work Synergistically to Promote Robust Mitotic Spindle Formation (A) Stills from movies of spindle formation in an EB1-GFP-expressing embryo injected with low levels of anti-DSpd-2, to dampen astral input. (B) Stills from movies of spindle reformation following cold treatment in an EB1-GFP-expressing embryo injected with low levels of anti-DSpd-2. (C) Line graph showing the fluorescence intensity over time in the region of mitotic chromatin following cold treatment in control (WT) embryos and embryos injected with anti-DSpd-2 antibodies. Reducing astral input results in increased generation of MTs around chromatin. Error bars represent SEM. n ≥ 20 spindles from at least three embryos. (D) EB1-GFP comets in control (WT) embryos, control embryos immediately following cold treatment, and embryos injected with anti-Dgt6 antibodies immediately following cold treatment. (E) Histogram of mitotic EB1-GFP comet length in embryos under different conditions. Curves to the right represent the histogram trends for comparison of data sets; the table below shows mean comet length and statistical significance between samples of interest. (F) Stills from time-lapse movies of spindle formation in an EB1-GFP-expressing embryo injected with both anti-Dgt6 and low levels of anti-DSpd-2. Mitotic spindles initially form from limited astral input, allowing measurement of EB1-GFP comet length, but intensity reduces and spindles eventually collapse. (G) Histogram of EB1-GFP comet velocity in embryos under different conditions. Curves to the right represent the histogram trends for comparison of data sets; the table below shows mean comet velocities of the samples of interest. Scale bars, 5 μm. See also Movie S10.

As the aforementioned pathway to spindle formation reflects the stripping of PCM from centrosomes rather than inactivation of centrosomal MT nucleating capacity per se, we instead injected lower concentrations of anti-DSpd-2 into EB1-GFP-expressing embryos (Figure 7A; Movie S10). This resulted in a significant reduction of centrosomally nucleated MTs, as determined by quantifying the EB1-GFP fluorescence emanating from centrosomes but without ectopic aMTOC formation (Figure S2). By injecting EB1-GFP embryos with this concentration of anti-DSpd-2, following cold treatment, we were able to assess the effect of specifically reducing astral input on chromatin-directed MT generation. It appeared to us that, during the initial stages of regrowth, a reduction of centrosomal EB1-GFP led to an increase in the intensity of EB1-GFP comets in the vicinity of the chromosomes (Figure 7B and Movie S10; compare with Figure 1G and Movie S2). Measurement of this phenomenon confirmed that, although the final steady-state nucleating capacity of chromatin was similar in the presence or absence of anti-DSpd-2, initial MT nucleation around chromosomes was dramatically increased in comparison to control embryos (Figure 7C).

To explore whether an inverse relationship exists between chromatin- and centrosomally nucleated MTs, we revisited our time-lapse movies of EB1-GFP dynamics during spindle formation in embryos lacking chromosome-generated MTs. We noticed that the length of the EB1 comets emanating from the centrosomes in anti-Dgt6-injected, cold-treated embryos appeared to be increased in comparison to the length of those from control cold-treated embryos (Figure 7D). We therefore undertook a quantitative analysis of EB1-GFP comet length after cold treatment in control, *mars*^*p*^ *(d-hurp)*, anti-Dgt6-, and anti-DSpd-2-treated embryos. This confirmed that a reduction of MT generation either from chromatin (*mars*^*p*^) or from centrosomes (anti-DSpd-2), or both (anti-Dgt6), causes an increase in EB1-GFP comet length on the remaining astral MTs (Figure 7E). This was especially pronounced in embryos injected with anti-Dgt6 antibodies and could be further enhanced by coinjecting anti-DSpd-2 and anti-Dgt6 antibodies together (Figure 7E; Movie S10). In the latter case, EB1 comets were, on average, four times the length of those in control embryos (4.76 μm versus 0.906 μm). Interestingly, this reduction of astral MT nucleation, together with the complete cessation of Augmin-driven MT generation, precluded the formation of stable bipolar spindles; EB1-GFP intensity reduced over time (Figure 7F), and initial regular spacing between centrosomes degenerated over time with neighboring asters collapsing on to each other (Movie S10). Thus, stable bipolar spindle formation requires a minimum number of MTs.

As the length of EB1-GFP comets is related to the dynamic properties of MTs (Bieling et al., 2007), we measured the growth rate of the astral MTs under the aforementioned conditions (Figure 7G). There was indeed a direct correlation between the length of the EB1-GFP signal on astral MTs and the rate at which they grew; EB1 comet velocity in cold-treated embryos injected with anti-Dgt6 was, on average, three times that of control embryos (0.645 μms^−1^ versus 0.256 μms^−1^). However, we did not observe a further increase in velocity upon concomitant disruption of Augmin and dampening of centrosomal nucleation (anti-Dgt6 and anti-DSpd2 together), suggesting a maximal velocity of MT growth in the early embryo of ∼0.6 μms^−1^. Nonetheless, our analysis confirms that, as the number of MTs in the mitotic embryo decreases, the remaining astral MTs increase their dynamic behavior. Together, these results show that the removal of one MT-generating pathway during spindle formation leads to a concurrent and synergistic increase in another.

## Discussion

Our experiments demonstrate that the mature mitotic spindle in *Drosophila* embryos, far from being formed via a single MT-generating pathway dependent on centrosomally derived MTs, is composed of MTs whose origins are, or can be, diverse. By disrupting the molecular basis of the individual pathways, we have assessed their relative contributions to spindle formation. In doing so, we demonstrate the underlying flexibility inherent within the system, in which removal of one MT-generating pathway causes the cell to respond by increasing its use of another.

The evidence supports a model in which normal, cycling embryonic centrosomes are preprimed with PCM and γ-Tubulin and exposed to α/β Tubulin dimer prior to the onset of mitosis so that they, together with amplification via Augmin, nucleate enough astral MTs to capture kinetochores quickly and efficiently—within 30 s of NEB. In contrast, the chromatin, which is not exposed to α/β Tubulin dimer or Augmin until after NEB, cannot participate in MT generation to any significant extent. However, by reversing MT nucleation after mature spindle formation through cold treatment, “rebooting” the system in midmitosis when both centrosomes and mitotic chromatin are equally exposed to Tubulin and Augmin, and quantitatively analyzing MT regrowth, we have shown that a chromatin-dependent pathway exists and, indeed, dominates over the centrosomes that are still present. This is not due to redistribution of γ-Tubulin from centrosomes, as γ-Tubulin-GFP intensity is not reduced at the centrosome in cold-treated embryos. Instead, it appears to be a consequence of sequestering and activating the SAF, D-HURP, around mitotic chromatin. Interestingly, in contrast to some other biological systems, including *Drosophila* S2 cells, the predominant site of new MT growth following cold treatment is not restricted to kinetochores (Torosantucci et al., 2008, Bucciarelli et al., 2009, Maiato et al., 2004) but occurs throughout the region of the mitotic chromatin. Human HURP generates and stabilizes MTs in a Ran-dependent manner (Silljé et al., 2006, Koffa et al., 2006). In the *Drosophila* embryonic scenario, we envisage that cold treatment of mitotic embryos after NEB leads to cell cycle arrest in which mitotic kinases and the Ran gradient are fully active, allowing the association of D-HURP with condensed chromatin where it nucleates short MT seeds. Subsequent removal of the temperature restriction will provide the necessary conditions for MT growth. That the chromatin-dependent pathway is also part of the normal complement of spindle-forming pathways in cycling embryos, but that its input is limited until later in mitosis, is supported by our observations that removal of D-HURP in cycling embryos results in shorter mature spindles that have a higher likelihood of failing in chromosome segregation.

In addition to astral and chromatin-dependent MT generation, we have revealed an alternative pathway to spindle formation. A failure to stably incorporate either DSpd-2 or Cnn to the centrosome results in cytosolic MT asters that coalesce into mature bipolar spindles. These aMTOCs are quite distinct from chromatin-dependent MTs, appearing within 10 s following NEB in regions of the cytoplasm devoid of chromosomes, and are qualitatively similar to those reported for acentriolar *Drosophila* cell lines (Moutinho-Pereira et al., 2009) and mouse oocytes (Brunet et al., 1998, Schuh and Ellenberg, 2007). They may, therefore, reflect a general mechanism of animal cell spindle formation in the absence of functioning centrosomes, where the nucleation and organization of MTs are achieved through concentration of nucleating activity at multiple cytosolic sites and bipolarity follows through their interaction and self-organization.

We have also provided clear evidence that all three pathways to spindle formation (centrosomal, chromatin, and aMTOC-driven) are dependent on a fourth: Augmin. Together with recent work demonstrating that new MTs can be produced in an Augmin-dependent manner using preexisting ones generated in vitro in *Xenopus* egg extracts (Petry et al., 2013), our evidence suggests that this conserved protein complex, once active, works on all existing mitotic MTs. However, whereas in *Xenopus* extracts, TPX2 is required for Augmin-generated MTs (Petry et al., 2013), our in vivo analysis of spindle formation in the absence of either D-TPX2 (using *mei-38* null mutants) or D-HURP supports a model in which D-HURP is the dominant chromatin-directed MT nucleator in *Drosophila* embryos, generating MT seeds that can then be amplified by Augmin. This likely reflects either a difference in function between the *Drosophila* and *Xenopus* proteins—for example, D-TPX2 shares homology with TPX2 only in its C-terminal domain and does not possess elements such as Aurora A targeting (Goshima, 2011)—or a difference in the usage of TPX2- and HURP-dependent pathways by different biological systems.

Our work also demonstrates that astral MT nucleation is dramatically reduced in cycling embryos lacking Augmin, in a D-TPX2- and D-HURP-independent manner. Under these conditions, the remaining astral MTs are eventually able to search and capture kinetochores, producing kinetochore-kMT interactions that allow Rod poleward streaming. However, the spindle assembly checkpoint remains unsatisfied, and Rod-GFP movement is perturbed, suggesting that some aspect of the interaction is incorrect. One possibility is that Augmin binds to and amplifies the initial kMTs, resulting in stable kMT bundles that can stream Rod poleward. Alternatively, the effect on the checkpoint may reflect the requirement of Augmin for generating many short non-kMT spindle MTs. It has recently been demonstrated that the viscoelastic properties of the *Xenopus* spindle—and its ability to transmit force as a unit—can be altered by reducing the density of such short, non-kMTs (Shimamoto et al., 2011). It is therefore possible that Augmin-dependent non-kMTs transmit force exerted on individual kinetochores by kMTs throughout the spindle as part of a spindle-scale sensing mechanism, intrinsically linked to the checkpoint. Whatever the molecular mechanism at work, our study supports a model in which Augmin binds indiscriminately to preexisting MTs to generate the bulk of the embryonic mitotic spindle, placing Augmin at the heart of MT generation during spindle formation. Interestingly, this scenario was predicted by mathematical models of *Drosophila* embryonic spindle organization, generated approximately 10 years ago (Brust-Mascher et al., 2004). In order for their model to recapitulate the dynamics of the anaphase spindle, the authors required the presence of multiple short MTs with origins that were distinct from centrosomes. Augmin fulfills such a role and, as such, incorporating its precise mode of action into future models of *Drosophila* spindle dynamics may well reveal additional features of spindle formation.

Although clearly essential for robust spindle formation in mitotic systems, Augmin and, indeed, γ-Tubulin have been shown to be dispensable for the bulk of MTs that form the initial *Drosophila* female meiosis I spindle (Colombié et al., 2013, Hughes et al., 2011). Instead, the *Drosophila* oocyte appears to rely on the Chromosomal Passenger Complex (CPC), the MT-stabilizing protein Minispindles, and the crosslinking motor Subito to organize stable cytoplasmic MTs, generated prior to meiosis onset, into an initial spindle structure (Jang et al., 2007, Cesario and McKim, 2011, Radford et al., 2012). This likely reflects the peculiarity of the pathways regulating formation of the meiotic spindle in this system. Nonetheless, it does suggest yet additional mechanisms by which a bipolar spindle can form, further highlighting the robustness of this structure.

Finally, importantly, we have shown that a reduction of astral input to spindle formation leads to an increase in chromatin-dependent MT generation, while removal of chromatin- or Augmin-dependent MT generation results in an increased accumulation of EB1-GFP at MT plus ends and an increase in the growth rate of the remaining astral MTs. The effect on EB1-GFP accumulation is accentuated if the number of remaining astral MTs is further reduced. These results suggest a coordinated and synergistic cellular response to perturbing mitotic MT-generating pathways. We do not know whether the increase in astral MT dynamics is a passive response to availability of resources, such as Tubulin.GTP dimer, or an active self-regulation, driven by monitoring of MT generation by the cell. In the simplest (passive) scenario, removing the MTs generated by one pathway could result in an increase in the available local concentration of Tubulin.GTP, shifting the dynamic equilibrium of remaining MTs further toward growth. Although in vitro studies have shown that increasing the concentration of Tubulin in solution increases MT growth rates (Walker et al., 1988) and that this correlates with increased accumulation of EB1 at the growing tips (Bieling et al., 2007), the high diffusion rate of Tubulin-GFP in the early embryo essentially rules out local depletion of resources close to individual MT tips as a source of variability (Hallen et al., 2008). Therefore, if resource depletion is responsible for limiting MT growth, it must be a global (spindle-scale) depletion. However, the increase in EB1 comet length and MT dynamics that occurs upon the loss of MT-generating pathways was measured in the early stages of spindle regrowth. At similar time points in embryos possessing all MT-generating pathways, the EB1 fluorescence (i.e., MT growth) in the region of the chromatin continues to increase dramatically over the following 2 min (compare Figure 5B with Figure 1G). Therefore, at these early time points, Tubulin.GTP, EB1, or any other cytoplasmic molecule that stimulates MT growth, cannot be depleted and therefore cannot be limiting growth. This leaves open the intriguing possibility that the cell somehow actively monitors the overall level of MT generation during spindle formation and alters flow through available pathways accordingly. Given this possibility, an important future goal will be to identify MAPs whose association with MTs changes upon inhibition of particular MT-generating pathways.

In summary, by revealing the presence of all the major mitotic MT-generating pathways described in animal cells within a single system, the *Drosophila* syncytial embryo, and by demonstrating a coordinated regulation between them, our work highlights the remarkable flexibility inherent in mitotic spindle formation. It implies that the key to building a successful spindle lies in activating a set of MT generators that together provide sufficient MTs to allow crosslinking and movement in relation to one another, regardless of how and where the MTs were initially generated. By subsequently limiting the nucleation and growth of these MTs to balance depolymerization, a steady-state spindle of defined length and physical properties is ultimately formed. Understanding the way in which a cell determines such a “Goldilocks zone” of MT generation will undoubtedly help us to understand the overall self-regulation of this fundamental cellular structure.

## Experimental Procedures

### Fly Stocks

The *d-tpx2* line, y*mei-38*^*1*^*w/y[+]Y/C(1)DX*, was a gift from Kim McKim (Wu et al., 2008); *EP(2)2477* (*mars*^*p*^), the *d-hurp* line, was a gift from Daimark Bennett (Tan et al., 2008). The centrosomin line, *cnn*^*hk21*^, was obtained from Bloomington Stock Center, while the *cnn*^*mfs7*^ line was a gift from Jordan Raff (Lucas and Raff, 2007). Fluorescent transgenes used were as follows: α-Tubulin-GFP and Histone-H3-RFP (Bloomington Stock Center); EB1-GFP and γ-Tubulin-GFP (gifts from Sharyn Endow) (Liang et al., 2009, Hallen et al., 2008); Rod-GFP (a gift from Roger Karess); Msd1-GFP (Wainman et al., 2009); GFP-Mars (a gift from Daimark Bennett) (Tan et al., 2008); and GFP-Mei-38. GFP-Mei-38 was generated by cloning the full-length *mei-38* cDNA into the vector pPGW via pENTR/D/TOPO (Invitrogen). The plasmid was injected into *w*^*1118*^ embryos by Bestgene. Expression of Msd1-GFP, GFP-Mars, and GFP-Mei-38 was driven using Maternal-α-Tubulin VP16 GAL4 (Bloomington Stock Center).

### Microscopy

Imaging was performed on a Visitron Systems Olympus IX81 microscope with a CSO-X1 spinning disk using a UPlanS APO 1.3 NA (Olympus) 60× objective. Embryos 1–2 hr old were manually dechorionated, aligned in heptane glue on 22 × 50 mm coverslips, and covered with a 1:1 mixture of Halocarbon oil 700 and Halocarbon oil 27) (Sigma). Imaging was performed with 400 ms exposure per slice, with five slices per stack and a constant room temperature of 22°C. Embryos were injected using an Eppendorf Inject Man NI 2 and Femtotips II needles (Eppendorf). The anti-DSpd-2 and anti-Dgt6 antibodies were suspended in injection buffer (100 mM HEPES, pH 7.4, and 50 mM KCl), centrifuged at 13,500 × g for 20 min, and injected at a concentration of either 6 mg/ml (anti-Dgt6 or anti-DSpd-2, high concentration) or 1 mg/ml (anti-DSpd-2, low concentration). For cold-treatment assays, single embryos were imaged until metaphase was reached, at which point they were placed in 50 mm ice-cold Petri dishes and covered with 4°C Halocarbon oil. Following 90 min on ice, embryos were reimaged, with 30 s typically expiring between removal from 4°C and the initiation of imaging. In some cases, embryos were removed following 75 min on ice, injected with antibody, and then placed on ice for another 15 min prior to imaging.

### Image Processing and Analysis

Image processing and analysis was performed on FIJI. Fluorescence loss caused by bleaching was corrected using the Bleach Corrector macro (developed by Kota Miura, European Molecular Biology Laboratory). Automated spindle tracking was achieved using custom image processing and object tracking algorithms. Briefly, scale-space filtering and object classification based on ellipse fitting allowed spindles to be detected. Next, centrosomes, also detected using scale-space analysis and image statistics based thresholding, were tracked in pairs using a modified Jonker-Volgenant (Global Nearest Neighbors) algorithm. Last, tracked spindle pairs were used to automatically generate many kymographs, which were combined within data sets to produce averages. Each experimental condition was undertaken on at least three independent occasions, showing qualitatively similar results. Composite kymographs shown are from single embryos, generated from between 4 and 13 spindles. All measurements and analyses were performed on time courses with maximum projected z stacks. The directionality of EB1-GFP comets emanating from the center of the spindle in cold-treated embryos was tracked using FIJI’s Manual Tracking plug-in, with the original coordinates of the comet normalized to 0,0. All spindles were rotated so that centrosomes lay on the x axis. Measurements were taken using ten spindles in total from three embryos. A χ^2^ analysis was undertaken to assess correlations in the distribution of the comet directions (p = 0.874). Spindle length comparisons between cycling control, *d-tpx2/mei-38*^*1*^, and *mars*^*p*^ embryos were undertaken by manually drawing a line from the center of each pair of centrosomes 10 s prior to onset of anaphase. At least five embryos from each condition were measured, with at least ten spindles measured per embryo. The p values were determined by pooling the spindle lengths from individual line. All p values are between control and *d-tpx2/mei-38*^*1*^ and *mars*^*p*^ mutant embryos. SD was determined using the pooled spindle lengths, while SEM was determined using the total number of embryos measured. To compare astral MT nucleation in control embryos, anti-DSpd-2-injected embryos, *d-hurp* embryos, and embryos injected with α-Dgt6 antibody, the relative centrosomal fluorescence for a specific time point was ascertained by subtracting the fluorescence of a circular region of interest (ROI) with a 1.5 μm radius from that of a circular ROI with a 3 μm radius (the center of both ROIs being the centrosome center), with this value divided by the mean length of EB1-GFP comet lengths for the time point in question. Centrosomes from three embryos were measured for each condition, with 22 (±8) centrosomes counted for each condition. At least 25 measurements for EB1-GFP comet length were taken for each time point for each embryo, with the mean comet length calculated for each embryo. Centrosomal fluorescence was divided by the mean comet length per embryo and SEM calculated, based on the number of centrosomes. To compare chromatin-mediated MT nucleation between control and anti-DSpd-2-injected cold-treated embryos, an ROI was drawn around the area of chromatin-mediated MT nucleation at 30 s following cold recovery, and the fluorescence was measured and tracked back to the first time-lapse frame. These data sets were normalized against internal background (normalized fluorescence = fluorescence of ROI − [background fluorescence × size of ROI]). EB1-GFP comet velocity was measured using Fiji’s Manual Tracking plug-in. EB1-GFP comet length was measured by drawing an ROI line from the visible ends of comets within the first 10 s following cold treatment in cold recovery samples and within the first 10 s of internuclear MT influx following NEB in cycling samples. The number of comets measured for both length and velocity for each sample was between 250 and 1,110, with comets from at least three embryos measured for each condition. The SD and confidence values were determined using n as the number of comets measured, while the p value was determined by pooling comet measurements from a single condition together from multiple embryos. All data analyses were performed on Excel (Microsoft).

## Supplemental Information

Document S1. Figures S1 and S2

Movie S1, Related to Figure 1. Spindles Form by Using an “Outwards-In” Mechanism in *Drosophila* Syncytial Embryos Mitosis visualized in Tubulin-GFP (green) and Histone-RFP (red) (left) and EB1-GFP (right) expressing syncytial embryos from nuclear envelope breakdown (NEB), as defined by the influx of soluble Tubulin-GFP/EB1-GFP into the nuclear space, until telophase. Spindles form in an “outwards-in” fashion. The field of view for each movie is 72.78 μm × 72.78 μm.

Movie S2, Related to Figure 1. MTs Are Nucleated around Chromatin following Cold Treatment, with Spindles Forming in an “Inwards-Out” Fashion Spindle formation visualized in Tubulin-GFP (green) and Histone-RFP (red) (left) and EB1-GFP (right) expressing syncytial embryos. MTs are clearly seen emanating from the region of condensed chromatin between centrosomes. The field of view for each is 32.36 μm × 32.36 μm.

Movie S3, Related to Figure 2. GFP-D-HURP Is Nuclear in Interphase before Localizing to Centrosomally Generated MTs GFP-D-HURP localization during syncytial embryonic mitosis. Chromatin and nuclear localization is visible prior to NEB. Upon NEB, GFP-D-HURP relocalizes to spindle MTs but not to centrosomes or asters. The field of view is 53.11 μm × 33.54 μm.

Movie S4, Related to Figure 3. The Mitotic Spindles of *d-tpx2* and *d-hurp* Mutant Embryos Are Short and Exhibit Defects *d-tpx2* cycle 11 embryo (left) and *d-hurp* cycle 12 embryo (right) both expressing EB1-GFP. In the absence of D-TPX2, spindles are visibly shorter than their wild-type counterparts (not shown) and barrel-like in appearance. *d-hurp* spindles also appear short. The field of view contains nucleus-less centrosomal pairs, a sign of nuclear fall-in resulting from defects in previous mitotic divisions. The field of view for each is 58.80 μm × 58.80 μm.

Movie S5, Related to Figure 3. Chromatin-Mediated MT Nucleation Is Absent in *d-hurp* Embryos following Cold Treatment Mitotic spindle formation in *d-tpx2* (left) and *d-hurp* (right) mutant syncytial embryos expressing EB1-GFP following cold treatment. Some chromatin-mediated MT nucleation is visible in *d-tpx2* embryos, but bipolar spindles often fail to form, with monopolar or collapsed spindles frequently resulting. In contrast, *d-hurp* embryos do not show chromatin-mediated MT nucleation, but bipolar spindles eventually form, apparently exclusively from centrosomally derived astral MTs. The field of view for each is 45.26 μm × 45.26 μm.

Movie S6, Related to Figure 4. Interfering Antibody Injections against Augmin Result in Delayed Spindle Formation and Weak, Long Spindles Mitotic spindle formation in syncytial embryos expressing Tubulin-GFP (green) and Histone-RFP (red) (left) and EB1-GFP (right) following injections with interfering antibodies raised against the Augmin subunit Dgt6. Astral MT nucleation is reduced and delayed in comparison to control embryos (Movie S1). Bipolar mitotic spindles form, apparently exclusively from centrosomally derived astral MTs, with the mature spindles arresting, possessing thick MT bundles, weak spindle density, and longer spindles. The field of view for both is 72.56 μm × 72.56 μm.

Movie S7, Related to Figure 4. Poleward Rod-GFP Streaming Is Present in Embryos Injected with α-Dgt6 Antibodies but Is Abnormal Mitosis in a control Rod-GFP-expressing syncytial embryo (left) and Rod-GFP-expressing embryo injected with α-Dgt6 antibodies (right). In control embryos, Rod-GFP accumulates at the kinetochores during prometaphase and streams toward the poles upon MT-kinetochore attachment. The intensity of Rod-GFP at the kinetochores continues to decrease throughout metaphase. By anaphase onset, streaming has stopped and the weak kinetochore-associated Rod-GFP can be seen segregating to poles with the chromosomes (time course finishes at 162 s). In Rod-GFP-expressing embryos injected with α-Dgt6 antibodies, Rod-GFP accumulates at the kinetochores following NEB, but streaming is delayed. Once streaming does occur, it appears asymmetrical and abnormal, with Rod-GFP movement both toward and away from the poles. Kinetochore accumulation does not decrease over time, consistent with a metaphase arrest. The field of view for each is 39.67 μm × 39.67 μm.

Movie S8, Related to Figure 5. Augmin Is Required for Chromatin-Mediated MT Nucleation Mitotic spindle formation in syncytial embryos expressing Tubulin-GFP (green) and Histone-RFP (red) (left) and EB1-GFP (right), injected with α-Dgt6 antibodies, following cold treatment. Unlike control cold-treatment recovery, in which spindle formation occurs “inwards-out” with chromatin as the major site of MT generation (Movie S2), MT nucleation in the vicinity of chromatin is absent and bipolar spindles form apparently exclusively from centrosomally derived astral MTs. The field of view for each is 42.75 μm × 42.75 μm.

Movie S9, Related to Figure 6. Perturbation of PCM Components DSpd-2 and Cnn Results in Augmin-Dependent aMTOC-Mediated Spindle Formation Mitotic spindle formation in EB1-GFP-expressing *cnn*^*mfs7*^*/cnn*^*hk21*^ embryo (left), EB1-GFP expressing control embryo injected with high concentration of antibody raised against DSpd-2 (center), and an EB1-GFP-expressing *cnn*^*mfs7*^*/cnn*^*hk21*^ embryo injected with α-Dgt6 antibody (right). In both the *cnn*^*mfs7*^*/cnn*^*hk21*^ embryo and the α-DSpd-2-injected embryo, aMTOCs, rather than definitive centrosomes, are visible throughout interphase. Upon NEB, these aMTOCs appear as the dominant sites of MT nucleation. Bipolar mitotic spindle formation occurs in an “outwards-in” manner. In contrast, although interphase aMTOCs are also present in the *cnn*^*mfs7*^*/cnn*^*hk21*^ embryo injected with α-Dgt6 antibody, upon NEB aMTOCs decrease in EB1-GFP fluorescence and spindle formation fails to occur. The field of view for each is 37.84 μm × 37.84 μm.

Movie S10, Related to Figure 7. Reducing MT Nucleation from Centrosomes via Injection of Low Concentrations of Interfering α-DSpd-2 Antibody Increases Chromatin-Mediated MT Nucleation in an Augmin-Dependent Manner Mitotic spindle formation in an EB1-GFP-expressing syncytial embryo injected with low concentrations of α-DSpd-2 antibody (left). Centrosome-driven bipolar spindles form. The field of view is 49.34 μm × 49.34 μm. Mitotic spindle formation in an EB1-GFP-expressing embryo cold treated and then injected with low concentration of α-DSpd-2 (center). Astral MT input to spindle formation is limited, while EB1-GFP fluorescence in the vicinity of mitotic chromatin is increased, compared to control embryos. The field of view is 61.28 μm × 61.28 μm. An EB1-GFP-expressing embryo, injected with both α-Dgt6 antibodies and low concentration of α-DSpd-2 antibodies, following cold treatment (right). Residual astral MTs emanate from the centrosomes, but these are insufficient to maintain spindle bipolarity. The field of view is 66.01 μm × 66.01 μm
