# Supplementary material for: Synergy between Multiple Microtubule-Generating Pathways Confers Robustness to Centrosome-Driven Mitotic Spindle Formation
Source: Dev Cell. 2014 Jan 13;28(1):81–93. doi: 10.1016/j.devcel.2013.12.001 (PMC3898610; doi:10.1016/j.devcel.2013.12.001)
Supplement: Document S1. Figures S1 and S2 [file mmc1.pdf]

Developmental Cell, Volume *28*

Supplemental Information

Synergy between Multiple Microtubule-Generating Pathways Confers Robustness to Centrosome-Driven Mitotic Spindle Formation

Daniel Hayward, Jeremy Metz, Claudia Pellacani, and James G. Wakefield

Supplemental Figures

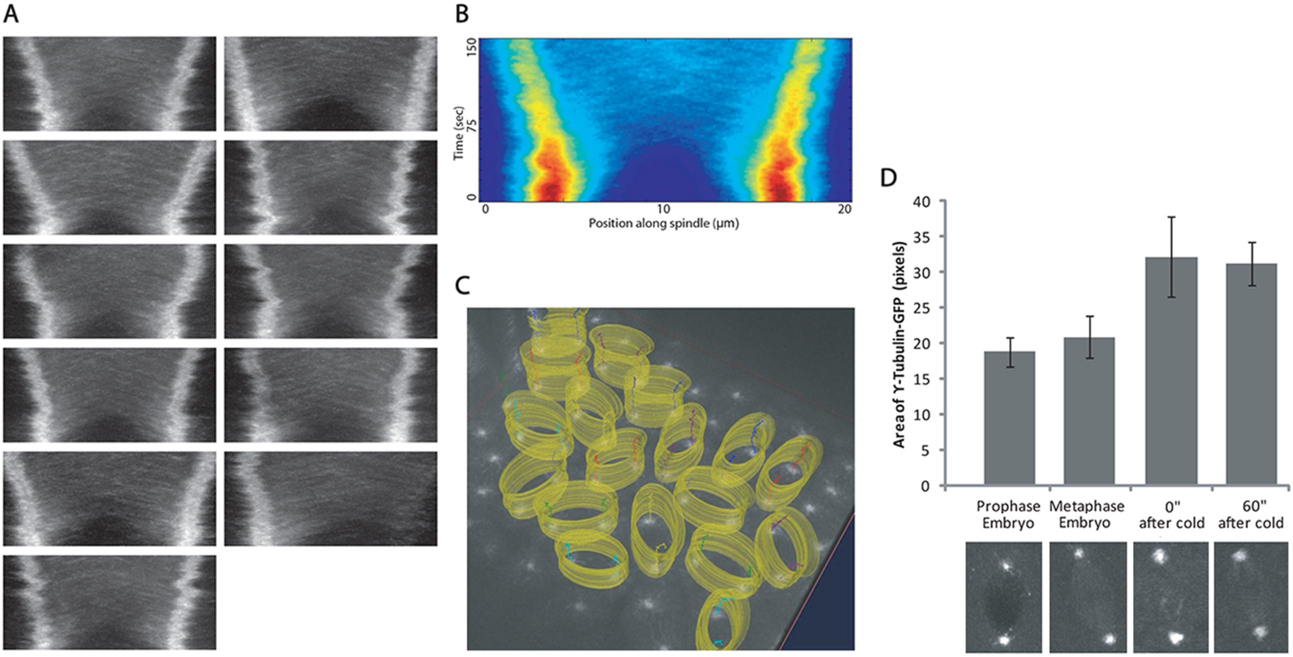

Figure S1, related to Figure 1. Quantitative analysis of MT generation and γ-Tubulin localisation in *Drosophila* embryos. (A) Kymographs of EB1-GFP fluorescence from individual mitotic spindles within a single embryo. (B) Composite kymographs are formed from compressed multiple kymographs and visualised using "heat-map" intensity profiles. (C) A "snap-shot" generated by the automated tracking algorithm, which identifies and segments individual mitotic spindles, prior to their analysis (see Experimental Procedures). (D) Bar chart showing the average area of γ-Tubulin-GFP fluorescence at the centrosomes in prophase embryos, metaphase embryos (60 seconds after NEB), cold treated metaphase embryos immediately after recovery (0”) and cold treated metaphase embryos 60 seconds following cold treatment recovery. Shown below is γ-Tubulin-GFP in representative spindles of each condition. Error bars represent S.E.M with at least 8 centrosomes per embryo from 3 embryos measured for each condition. n is based on the number of embryos used.

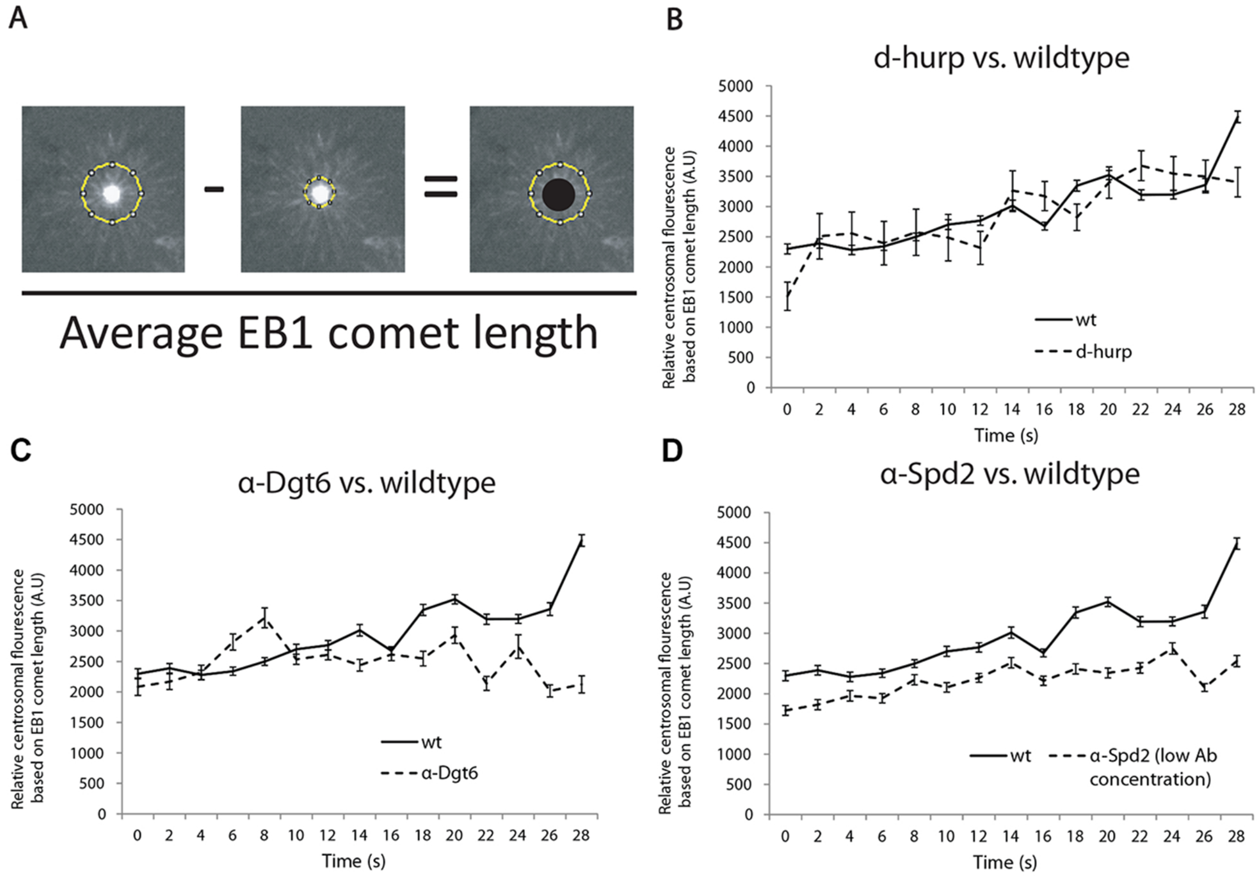

Figure S2, related to Figure 3. Quantification of centrosome-mediated MT nucleation in wild type, d-hurp mutant, anti-Dgt6 injected and anti-DSpd-2 injected (low concentration) embryos, expressing EB1-GFP. (A) A diagrammatic representation of the method used to measure centrosome-mediated MT nucleation. Briefly, a 6µm circular ROI was drawn around a centrosomal area, with the centrosome in the centre and the total fluorescence measured. From this, the total fluorescence of a 3µm circular ROI drawn around the centrosome was subtracted (with both values being normalised against background fluorescence). The resulting value was then divided by the mean average length of EB1-GFP comets within the sample to give the relative centrosomal fluorescence based on EB1-GFP comet length. (B-D) Line graphs showing the relative EB1-GFP fluorescence in the centrosomal area in the first 30 seconds following cold treatment, comparing control (wild type) embryos with *d-hurp* embryos (B), embryos that have been injected with α-Dgt6 antibodies (C) and embryos injected with a low concentration of α-DSpd-2 antibody (D). Error bars represent S.E.M.

Supplemental Movie Legends

Movie S1, related to Figure 1. Spindles form using an "outwards-in" mechanism in *Drosophila* syncytial embryos

Mitosis visualised in Tubulin-GFP (green) and Histone-RFP (red) (left) and EB1-GFP (right) expressing syncytial embryos from nuclear envelope breakdown (NEB), as defined by the influx of soluble Tubulin-GFP/EB1-GFP into the nuclear space, until telophase. Spindles form in an "outward-in" fashion. The field of view for each movie is 72.78µm x 72.78µm.

Movie S2, related to Figure 1. MTs are nucleated around chromatin following cold treatment, with spindles forming in an "inwards-out" fashion

Spindle formation visualised in Tubulin-GFP (green) and Histone-RFP (red) (left) and EB1-GFP (right) expressing syncytial embryos. MTs are clearly seen emanating from the region of condensed chromatin, between centrosomes. The field of view for each is 32.36µm x 32.36µm.

Movie S3, related to Figure 2. GFP-D-HURP is nuclear in interphase, before localising to centrosomally-generated MTs

GFP-D-HURP localisation during syncytial embryonic mitosis. Chromatin and nuclear localisation is visible prior to NEB. Upon NEB, GFP-D-HURP re-localises to spindle MTs, but not to centrosomes or asters. The field of view is 53.11µm x 33.54µm.

Movie S4, related to Figure 3. The mitotic spindles of *d-tpx2* and *d-hurp* mutant embryos are short and exhibit defects

d-tpx2 cycle 11 embryo (left) and *d-hurp* cycle 12 embryo (right) both expressing EB1-GFP. In the absence of D-TPX2, spindles are visibly shorter than their wild type counterparts (not shown) and barrel-like in appearance. *d-hurp* spindles also appear short. The field of view contains nucleus-less centrosomal pairs; a sign of nuclear fall-in resulting from defects in previous mitotic divisions. The field of view for each is 58.80µm x 58.80µm.

Movie S5, related to Figure 3. Chromatin mediated MT nucleation is absent in *d-hurp* embryos following cold treatment

Mitotic spindle formation in *d-tpx2* (left) and *d-hurp* (right) mutant syncytial embryos expressing EB1-GFP, following cold treatment. Some chromatin mediated MT nucleation is visible in *d-tpx2* embryos, but bipolar spindles often fail to form, with monopolar or collapsed spindles frequently resulting. In contrast, *d-hurp* embryos do not show chromatin mediated MT nucleation, but bipolar spindles eventually form, apparently exclusively from centrosomally-derived astral MTs. The field of view for each is 45.26µm x 45.26µm.

Movie S6, related to Figure 4. Interfering antibody injections against Augmin result in delayed spindle formation and weak, long spindles

Mitotic spindle formation in syncytial embryos expressing Tubulin-GFP (green) and Histone-RFP (red) (left) and EB1-GFP (right) following injections with interfering antibodies raised against the Augmin subunit Dgt6. Astral MT nucleation is reduced and delayed in comparison to control embryos (Movie S1). Bipolar mitotic spindles form, apparently exclusively from centrosomally-derived astral MTs, with the mature spindles arresting, possessing thick MT bundles, weak spindle density and longer spindles. The field of view for both is 72.56µm x 72.56µm.

Movie S7, related to Figure 4. Poleward Rod-GFP streaming is present in embryos injected with α-Dgt6 antibodies, but is abnormal

Mitosis in a control Rod-GFP-expressing syncytial embryo (left) and Rod-GFP-expressing embryo injected with α-Dgt6 antibodies (right). In control embryos, Rod-GFP accumulates at the kinetochores during prometaphase and streams towards the poles upon MT-kinetochore attachment. The intensity of Rod-GFP at the kinetochores continues to decrease throughout metaphase. By anaphase onset, streaming has stopped and the weak kinetochore-associated Rod-GFP can be seen segregating to poles with the chromosomes (time course finishes at 162 secs). In Rod-GFP-expressing embryos injected with α-Dgt6 antibodies, Rod-GFP accumulates at the kinetochores following NEB, but streaming is delayed. Once streaming does occur, it appears asymmetrical and abnormal, with Rod-GFP movement both towards and away from the poles. Kinetochore accumulation does not decrease over time, consistent with a metaphase arrest. The field of view for each is 39.67µm x 39.67µm.

Movie S8, related to Figure 5. Augmin is required for chromatin mediated MT nucleation

Mitotic spindle formation in syncytial embryos expressing Tubulin-GFP (green) and Histone-RFP (red) (left) and EB1-GFP (right), injected with α-Dgt6 antibodies, following cold treatment. Unlike control cold treatment recovery, where spindle formation occurs “inwards-out” with chromatin as the major site of MT generation (Movie S2), MT nucleation in the vicinity of chromatin is absent and bipolar spindles form apparently exclusively from centrosomally-derived astral MTs. The field of view for each is 42.75µm x 42.75µm.

Movie S9, related to Figure 6. Perturbation of PCM components DSpd-2 and Cnn result in Augmin dependent aMTOC mediated spindle formation

Mitotic spindle formation in EB1-GFP-expressing cnn^{mfs7}/cnn^{hk21} embryo (left), EB1-GFP expressing control embryo injected with high concentration of antibody raised against DSpd-2 (centre) and an EB1-GFP-expressing cnn^{mfs7}/cnn^{hk21} embryo injected with α-Dgt6 antibody (right). In both the cnn^{mfs7}/cnn^{hk21} embryo and the α-DSpd-2 injected embryo aMTOCs, rather than definitive centrosomes, are visible throughout interphase. Upon NEB, these aMTOCs appear as the dominant sites of MT nucleation. Bipolar mitotic spindle formation occurs in an “outwards-in” manner. In contrast, although interphase aMTOCs are also present in the cnn^{mfs7}/cnn^{hk21} embryo injected with α-Dgt6 antibody, upon NEB aMTOCs decrease in EB1-GFP fluorescence and spindle formation fails to occur. The field of view for each is 37.84µm x 37.84µm.

Movie S10, related to Figure 7. Reducing MT nucleation from centrosomes, via injection of low concentrations of interfering α-DSpd-2 antibody, increases chromatin mediated MT nucleation in an Augmin dependent manner

Mitotic spindle formation in an EB1-GFP-expressing syncytial embryo injected with low concentrations of α-DSpd-2 antibody (left). Centrosome-driven bipolar spindles form. The field of view is 49.34µm x 49.34µm. Mitotic spindle formation in an EB1-GFP-expressing embryo cold treated then injected with low concentration of α-DSpd-2 (centre). Astral MT input to spindle formation is limited, while EB1-GFP fluorescence in the vicinity of mitotic

chromatin is increased, compared to control embryos. The field of view is 61.28μm x 61.28μm. An EB1-GFP expressing embryo, injected with both α-Dgt6 antibodies and low concentration of α-DSpd-2 antibodies, following cold treatment (right). Residual astral MTs emanate from the centrosomes, but these are insufficient to maintain spindle bipolarity. The field of view is 66.01μm x 66.01μm
